# Ultrasound-Guided Access Reduces Vascular Complications in Patients Undergoing Catheter Ablation for Cardiac Arrhythmias

**DOI:** 10.3390/jcm11226766

**Published:** 2022-11-15

**Authors:** Leonie Foerschner, Nico Erhard, Stephan Dorfmeister, Marta Telishevska, Marc Kottmaier, Felix Bourier, Sarah Lengauer, Carsten Lennerz, Fabian Bahlke, Hannah Krafft, Florian Englert, Miruna Popa, Christof Kolb, Gabriele Hessling, Isabel Deisenhofer, Tilko Reents

**Affiliations:** Department of Electrophysiology, German Heart Center Munich, Technical University of Munich, Lazarettstr. 36, 80636 Munich, Germany

**Keywords:** catheter ablation, atrial arrhythmias, ventricular arrhythmias, ultrasound-guided vascular access, vascular access complications

## Abstract

Background: Femoral vascular access using the standard anatomic landmark-guided method is often limited by peripheral artery disease and obesity. We investigated the effect of ultrasound-guided vascular puncture (UGVP) on the rate of vascular complications in patients undergoing catheter ablation for atrial or ventricular arrhythmias. Methods: The data of 479 patients (59% male, mean age 68 years ± 11 years) undergoing catheter ablation for left atrial (*n* = 426; 89%), right atrial (*n* = 28; 6%) or ventricular arrhythmias (*n* = 28; 6%) were analyzed. All patients were on uninterrupted oral anticoagulants and heparin was administered intravenously during the procedure. Femoral access complications were compared between patients undergoing UGVP (*n* = 320; 67%) and patients undergoing a conventional approach (*n* = 159; 33%). Complication rates were also compared between patients with a BMI of >30 kg/m^2^ (*n* = 136) and patients with a BMI < 30 kg/m^2^ (*n* = 343). Results: Total vascular access complications including mild hematomas were *n* = 37 (7.7%). In the conventional group *n* = 17 (10.7%) and in the ultrasound (US) group *n* = 20 (6.3%) total vascular access complications occurred (OR 0.557, 95% CI 0.283–1.096). UGVP significantly reduced the risk of hematoma > 5 cm (OR 0.382, 95% CI 0.148, 0.988) or pseudoaneurysm (OR 0.160, 95% CI 0.032, 0.804). There was no significant difference between the groups regarding retroperitoneal hematomas or AV fistulas (*p* > 0.05). In patients with BMI > 30 kg/m^2^, UGVP led to a highly relevant reduction in the risk of total vascular access complications (OR 0.138, 95% CI 0.027, 0.659), hematomas > 5 cm (OR 0.051, 95% CI 0.000, 0.466) and pseudoaneurysms (OR 0.051, 95% CI 0.000, 0.466). Conclusion: UGVP significantly reduces vascular access complications. Patients with a BMI > 30 kg/m^2^ seem to particularly profit from a UGVP approach.

## 1. Introduction

Catheter ablation is increasingly used to treat patients with various cardiac arrhythmias [1]. Most ablation procedures require periprocedural oral anticoagulation treatment to minimize the risk of thrombus formation [1,2]. In patients with atrial fibrillation (AF), complications rates are reported between 6–8% [3]. Other studies show thromboembolic events in 0.7%, major bleeding events in 1.6% and minor bleeding events in 9.5% [4,5]. The incidence of vascular complications in patients undergoing ventricular tachycardia ablation is reported to be higher (4–6%) than in patients undergoing AF ablation (1–2%) due to femoral arterial access [6,7,8]. In patients with vascular disease or obesity, a standard anatomic-landmark-guided method for femoral access is often associated with multiple puncture attempts, inadvertent arterial puncture or unsuccessful cannulation resulting in vascular access complications [9,10]. Additionally, current literature shows that female gender and age can impact the incidence of vascular access complications [11].

To improve safety, the use of two-dimensional US has become standard practice in fields such as anesthesia and nephrology [12].

To date, conflicting data exist regarding the benefit of UGVP in patients undergoing catheter ablation for atrial and ventricular arrhythmias.

## 2. Methods

The data of 479 adult patients (59% male, mean age 68 years ± 11 years) undergoing catheter ablation (primary as well as redo ablations) between May and December 2020 for atrial or ventricular arrhythmias were analyzed.

Clinical data including type of arrhythmia, relevant medical history, oral anticoagulation (OAC), ablation strategy and periprocedural complications were derived from the center’s database.

Medical history included heart failure (ejection fraction ≤ 45%), CHA2DS2-VASc-Score, hypertension, diabetes, history of stroke/TIA, vascular disease, coronary artery disease (CAD), peripheral artery disease (PAD) and BMI. BMI >/<30 kg/m^2^ was prespecified for sub-group analysis. Arrhythmias were defined as paroxysmal or persistent atrial fibrillation (AF), atrial flutter or atrial tachycardia, as well as right and left premature ventricular contractions (PVC) or ventricular tachycardias (VT). Patients with ventricular arrhythmias or atrial tachycardias were only included if they were receiving oral anticoagulation (OAC) (e.g., because of concomitant AF or other indications for OAC).

All patients were on uninterrupted oral anticoagulants prior to catheter ablation (58% apixaban, 15% rivaroxaban, 15% edoxaban, 3.8% dabigatran and 7.7% vitamin-K antagonists) and heparin was administered by IV during the procedure targeting an ACT of >300 s. Although several studies reported no difference in complication rates between patients with and without previous OAC, we aimed to generate the most standardized condition for the study collective [12,13,14]. Femoral access complications were compared between two patient groups (US guidance, *n* = 320, 67%; or conventional approach, *n* = 159, 33%).

Our preprocedural management has been described previously [15]. All patients received a US of their groin vessels on the day before ablation to exclude major anomalies. No previous anomalies could be found before the ablation procedure. However, US diagnostics before the ablation procedure can also facilitate puncture conditions in the conventional group. Importantly, it should be mentioned that in our institution, a diagnostic US of groin vessels is conducted one day before the procedure by the angiological staff, and not by the EP staff, only to guarantee the possibility of puncturing the groin vessel. If anomalies or previous AV fistulas are found, alternative access (e.g., right jugular vein/left subclavian vein) is preferred.

Periprocedural patient management was identical in the US-guided and the conventional group.

## 3. Vascular Access by Conventional or UGVP Technique

Between May and June 2020, vascular access was obtained using the modified Seldinger technique, conventional surface anatomical landmarks and palpation of the femoral arterial pulse for femoral vein/arterial puncture.

Between November and December 2020, a real-time 2-dimensional vascular US (SonoSite S-II, Fujifilm SonoSite Inc., Bothell, WA, USA) was used to guide femoral venous or arterial access.

Between June and November, US was not yet used by default in our EP laboratories.

**Figure 1:**
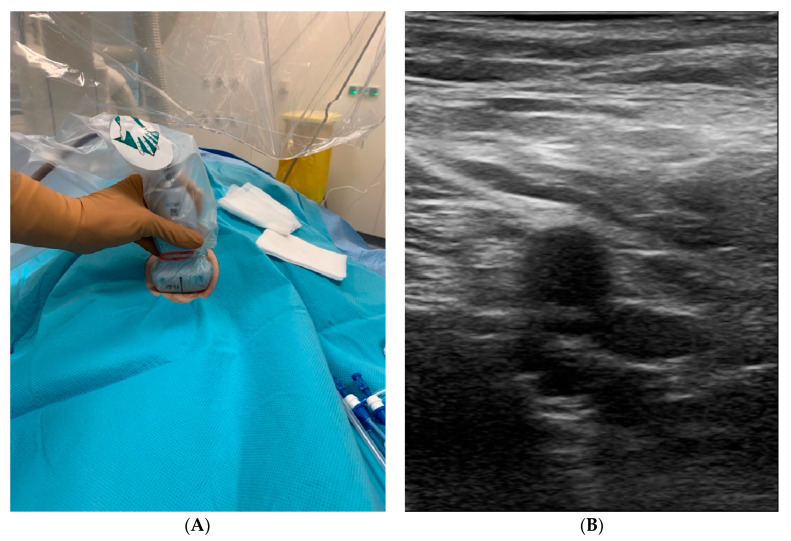
UGVP in the right groin. (**A**) The operator holds the probe while puncturing with the other hand. (**B**) The optimal site for puncture with the vein and artery visualized side-by-side (A: artery; V: vein).

An 8 MHz linear array ultrasonography transducer (US beam depth between 4 to 6 cm) was connected to the portable echocardiograph and covered with a sterile sleeve. After placement of the transducer at a 90-degree angle to the course of the vein at the transverse view (Figure 1), the femoral vein was differentiated from the artery by compression by the transducer, and vascular cannulation was performed under visualization of the needle passing into the vein and aspiration of venous blood into the syringe [16]. To avoid an incorrect puncture, access was gained above the bifurcation of the artery, where the vein and artery are placed next to each other, not above.

All operators were appropriately trained (>10 US-guided procedures) to qualify for the study.

## 4. Ablation Procedure and Periprocedural Management

The ablation procedures were performed as previously described by our group [15].

Heparin was administered by IV during the procedure, targeting an ACT of >300 s. At the end of the procedure, no protamine was given. In all cases, a purse-string suture was applied to venous puncture sites. Venous sheaths were removed directly after purse-string suture, and a groin compression bandage was applied for 2 h in patients with only venous sheaths. For arterial punctures, a vascular closure system (FemoSeal^®^, Angio-Seal^®^) was used. A groin compression bandage was additionally implemented for 6 h in these cases.

On the day, after the ablation procedure, all punctured groins were carefully clinically examined focusing on hematoma size and individual pain levels, as well as overall clinical presentation. All patients with mild hematoma or pain received a routine in-hospital US of the groin vessel on the first day post-ablation in order to rule out any vascular access complications.

## 5. Study Endpoints

Primary endpoints of the study were the occurrence of vascular complications including hematoma >5 cm, AV fistulas, pseudoaneurysms and retroperitoneal hematomas, treated conservatively or requiring intervention within the first 48 h after the procedure. Secondary endpoints were hemoglobin drop within the first 48 h after the procedure and procedure duration.

## 6. Statistical Analysis

Continuous variables in both groups are presented as mean ± standard deviation and compared by two-sided *t*-test for independent samples, by or Mann–Whitney U test, as appropriate. Fisher’s exact test or chi-square test was used for categorical variables. Risk reduction was calculated using the binary logistic regression for the overall collective as well as for the subgroup analysis (BMI </> 30 kg/m^2^, presence of vascular disease, arterial vs. venous puncture). All results were adjusted to differences in the baseline characteristics to avoid confounding. A *p*-value < 0.05 was considered significant. All analyses were performed using the SPSS 27.0 statistical package (SPSS Inc., Chicago, IL, USA).

## 7. Results

All baseline characteristics are presented in Table 1. International normalized ratio (INR) was significantly lower in patients with the conventional approach compared to patients with UGVP (1.18 ± 0.0.36 vs. 1.26 ± 0.48; *p*-value 0.009). For all other baseline characteristics, no significant differences between the groups were present.

In Table 2, types of arrhythmias are presented. No significant differences between the types of arrhythmias or arterial or venous puncture were shown (*p* > 0.05). All patients (also patients with ventricular tachycardias and atrial tachycardias) were under oral anticoagulants.

In Table 3, oral anticoagulation and antiplatelet agents before the procedure are shown.

In Table 4 procedural data are listed.

## 8. Peri-/Postprocedural In-Hospital Vascular Access Complications

Total vascular access complications within the first 48 h after the procedure are shown in Table 5.

One patient in the conventional group with an AV fistula was transferred to another hospital for vascular surgery on day three after the procedure. One patient in the US group with hemodynamic relevant AV fistula received a stenting of the right superficial femoral artery on day two after the procedure. One patient in the conventional group with retroperitoneal bleeding received a stenting of the right superficial external pudendal artery on day one after the procedure.

## 9. Peri-/Postprocedural In-Hospital Thromboembolic and Other Complications

Other peri-/postprocedural complications are shown in Table 6. One patient in the US group after left atrial tachycardia re-ablation was resuscitated, because of higher AV-block and received a pacemaker one day after the procedure. One patient in the conventional group generated ventricular fibrillation during the VT ablation and received CPR because of cardiogenic shock.

## 10. Risk Factors for Vascular Access Complications

All results for vascular complications were adjusted to the significant group differences for baseline characteristics. Significant risk factors for AV fistulas were vascular disease (OR 3.1; *p* = 0.007) and ventricular ablation procedures (OR 12.7; *p*-value 0.003).

A BMI > 30 kg/m^2^ (OR 4.3; *p*-value = 0.047) and ventricular ablation procedures (OR = 5.274; *p*-value = 0.049) were significant risk factors for pseudoaneurysms (Supplementary Table S1). INR at admission to the hospital had a significant impact on AV fistulas (OR 2.05; *p* = 0.022). Minimal ACT had a significant impact on incidence of pseudoaneurysms (OR 1.014; *p* = 0.010). After adjustment to minimal ACT, a significant reduction in the risk of pseudoaneurysms for patients with UGVP was still obtained (OR = 7.6; *p* = 0.027). Maximal and mean ACT as well as total heparin dose had no impact on the incidence of any vascular access complication (*p* > 0.05).

Additionally, age > 70 years and female sex had no significant impact on total vascular access complications, hematomas >5 cm, AV fistulas or pseudoaneurysms (*p* > 0.05) (see Supplementary Tables S2 and S3).

All other significant group differences for baseline characteristics (medication with apixaban or rivaroxaban, procedure duration, RF duration) had no significant impact on any vascular access complications (*p* > 0.05). Thus, these baseline and procedural data were not confounders for vascular access complications.

## 11. Reduction in the Risk of Vascular Access Complications in the Overall Collective and in the Subgroup Analysis

The subgroup analysis for patients with BMI > 30 kg/m^2^ is shown in Table 7.

UGVP led to a 95% reduction in hematomas >5 cm and pseudoaneurysms, and an 87% reduction in total vascular access complications. For AV fistulas and retroperitoneal hematomas, no significant risk reduction could be obtained in patients with BMI > 30 kg/m^2^.

In patients with BMI < 30 kg/m^2^ no significant reduction in the risk of hematoma, pseudoaneurysms or total vascular access complications was obtained.

## 12. Discussion

Our study demonstrated that UGVP reduces major vascular complications (hematoma >5 cm, pseudoaneurysms), particularly in patients with elevated BMI (>30 kg/m^2^).

In accordance with our findings, La Greca et al. demonstrated a significant risk reduction of 86% (1% vs. 7%; *p* = 0.004) in major vascular access complications using UGVP and intracardiac echocardiography during catheter ablation of atrial fibrillation. The combined approach also required less fluoroscopy time (−6 min) (median with US 14 min; IQR 8–12 vs. median without US 22 min; IQR 17-32; *p* < 0.001) and less radiofrequency time (median with US 1686 s; IQR 1367-1998 vs. median without US 1792 s; IQR 1390-2400; *p* = 0.012) [13]. In our study, we found a significant reduction of 18.5 min in procedure duration using UGVP. US also led to a reduction in radiofrequency time by 4.7 min. This may be caused by unsuccessful cannulation or inadvertent arterial puncture with consequent manual compression.

In a large single-center study, Sharma et al. showed that UGVP for patients undergoing EP procedures for various cardiac arrhythmias was associated with a significantly lower 30-day risk of vascular access complications (5.3% vs. 1.1%; *p* = 0.002). Increased age and non-US vascular access were risk factors for vascular access complications. In our study, especially in patients with elevated BMI, non-US vascular access was a risk factor for vascular access complications.

In the recently published prospective trial (ULTRA-FAST trial) with 320 consecutive atrial fibrillation ablation procedures, patients undergoing UGVP showed no significant difference in complication rates, but lower puncture time (288 vs. 369 s: *p* = 0.001), fewer inadvertent arterial punctures and unsuccessful cannulations, as well as fewer extra puncture attempts and higher first-pass success [10]. This effect was particularly evident in the subgroup of trainees. This indicates that trainees may have less understanding of groin anatomy and can benefit from UGVP. Yamagata also stated that anatomical difficulties with position of the femoral vein directly above the artery (65% of patients overlap with the artery in the anteroposterior plane [17]) can lead to inadvertent artery puncture and AV fistula. In this case, US can be of particular benefit.

Compared to the above-mentioned studies, our overall complication rate might seem to be higher. This is caused by the documentation of even minor complications (hematomas, mild AV fistulas and pseudoaneurysms without treatment) and the high frequency of US documentation in our center. However, we would like to point out that in our study all patients with mild hematoma or pain received a routine in-hospital US of the groin vessel on the first day post-ablation. To the best of our knowledge, post-ablation US is not commonly performed in most centers. Above-mentioned studies conducted US only in highly symptomatic patients or documented only major complications (AV fistulas or pseudoaneurysms with intervention) [13].

Major complications requiring intervention, in our study, only represent 0.4% (0.3% in the UGVP group and 0.6% in the conventional group), which is similar to the percentages reported in previous studies.

Therefore, we believe that the higher overall incidence of AV fistulas is mainly the result of a higher overall detection rate compared to previous studies, resulting in identifying more asymptomatic fistulas, which otherwise would not have been noticed.

Overall, UGVP is a safe method with a short learning curve (estimated at six patients) with a special benefit for trainees in preventing major vascular complications and improving workflow, as well as reducing procedure duration [12,18,19].

## 13. Periprocedural Anticoagulation, Intraprocedural Heparin Dose and ACT Levels

Similar to our findings, Tanaka-Esposito et al. showed in a large retrospective study of patients undergoing pulmonary vein isolation for atrial fibrillation a significant reduction in the risk of vascular complications despite a higher INR in the US group. An INR > 1.2 was associated with more vascular complications, which is comparable to our study [20].

In our study mean ACT, maximal ACT and total heparin dose showed no impact on the incidence of vascular access complications, while elevated INR and had an impact on the incidence of AV fistulas.

Interestingly, in our study, ACT values (min., max.) were significantly higher in the US group, while vascular complications were significantly more frequent in the conventional group.

This suggests an additional safety effect of the US group allowing for safe puncture/access even in patients at higher risk for bleeding.

Current literature demonstrates that continuation of warfarin at a therapeutic INR at the time of atrial fibrillation ablation without use of heparin or enoxaparin for bridging is a safe and efficacious periprocedural anticoagulation strategy [21,22]. Furthermore, the optimal INR range during uninterrupted periprocedural anticoagulation using warfarin is estimated to be narrow and has not been defined yet in current guidelines [23].

Unfractionated heparin is commonly administered after sheath insertion and continued to maintain an ACT longer than 250–350 s. Thus, intraprocedural anticoagulation schemes differ between centers. About 70% of centers routinely administer heparin before the transseptal puncture, when a transseptal approach is used [24,25]. In our center, ACT target values differ between 300 and 350 s.

Our findings, i.e., that mean ACT, maximal ACT and heparin dose during the ablation procedure had no major impact on the incidence of vascular access complications, indicate that quality of puncture (improved by US guidance) is probably more important than ACT values for the occurrence of vascular access site complications.

## 14. Risk Factors for Vascular Complications

The current literature focuses almost exclusively on risk factors *for overall complications* for patients undergoing atrial fibrillation ablation or ablation for ventricular tachycardias. In most studies, predictors for these overall complications were coronary artery disease and peripheral artery disease, as well as age > 50 years, chronic kidney disease, hypertension and female gender [11,26,27]. Additionally, an analysis of the Nationwide Inpatient Sample (2005–2013) shows that obesity is an independent risk factor for immediate post-ablation complications and higher costs for patients undergoing catheter ablation for atrial fibrillation [28,29].

However, to the best of our knowledge, data with conflicting results exist about the predictors of vascular complications after catheter ablation.

Dalsgaard et al. also showed that ACT levels had no impact of the incidence on vascular complications. His study indicates that any immediate hematoma after the procedure is the sole predictor for groin hematomas after the procedure. Thus, the purse-strig suture, which is routinely performed in our center, as well as arterial closure systems, can reduce the incidence of hematomas [30,31].

A higher BMI in our cohort was associated with a higher incidence of total vascular access complications, hematoma > 5 cm and pseudoaneurysms. The correlation between BMI and groin complications is controversial in the current literature. Although some studies state that the incidence of groin complications is independent of a patient’s weight, others show that a higher BMI (moderately high BMI ≥ 28 kg/m^2^–obesity class 3 BMI ≥ 40 kg/m²) might be a significant risk factor for the development of vascular access complications [32,33,34].

## 15. Limitations

The study is limited by its retrospective design. Due to multiple atrial and ventricular arrhythmia forms, the cohort is heterogeneous. Further randomized controlled trials are desirable to implement the findings in current practice and guidelines. To guarantee standardized bleeding conditions in this heterogenous group of cardiac arrhythmias, we only included patients on OAC in our study. However, several studies reported no difference in complication rates between patients with and without previous OAC [12,13,14]. This selection criteria might cause a potential bias in our study.

Furthermore, we conducted a US of the groin vessel in all patients before catheter ablation to exclude major anomalies. Although no anomalies could be found before the procedure, this could cause a potential selection bias in our study.

A comparatively high incidence of overall AV fistulas was observed in this study. However, we would like to point out that all patients with mild hematoma or pain received a routine in-hospital US of the groin vessel on the first day post-ablation. This is routine practice at our center. We believe that the higher overall incidence of AV fistulas is mainly the result of a higher overall detection rate compared to previous studies, resulting in identifying more asymptomatic fistulas, which otherwise would not have been noticed.

## 16. Conclusions

Vascular complications are the most common complications in patients undergoing catheter ablation for cardiac arrhythmias. Peripheral artery disease, obesity and ventricular ablation procedures are predictors for vascular complications. UGVP is a safe method with a short learning curve (estimated at six patients) with special benefits for trainees to prevent major vascular complications and to improve workflow, as well as to reduce procedure duration. Patients with a BMI > 30 kg/m^2^ received the highest benefit from US guidance.

## Figures and Tables

**Table 1:** Baseline characteristics.

	Total *n* = 479	Conventional *n* = 159	US *n* = 320	*p*-Value
Age (years)	68.5 ± 11.3	68.2 ± 11.0	68.6 ± 11.4	0.563
Gender (male)	281 (58.7%)	99 (62.3%)	182 (56.9%)	0.259
BMI (kg/m^2^)	27.6 ± 5.0	28.1 ± 5.3	27.3 ± 4.8	0.139
BMI > 30 kg/m^2^	136 (28.4%)	52 (32.7%)	84 (26.3%)	0.140
Hypertension	333 (69.5%)	114 (71.1%)	219 (68.4%)	0.465
Diabetes	69 (14.4%)	21 (13.2%)	48 (15%)	0.599
History of stroke/TIA	43 (9%)	14 (8.8%)	29 (9.1%)	0.926
CAD, PAD	145 (30.3%)	55 (34.6%)	90 (28.1%)	0.147
First ablation	219 (45.7%)	76 (47.8%)	143 (44.7%)	0.520
CHA2DS2-VASc-Score	2.96 ± 1.62	2.94 ± 1.61	2.97 ± 1.62	0.856
GFR ^1^	73.1 ± 20.6	72.7 ± 20.2	73.3 ± 20.8	0.769
Hb mg/dL	14.0 ± 1.6	14.1 ± 1.3	14.0 ± 1.7	0.359
Serum Creatinine mg/dL	1.1 ± 0.3	1.1 ± 0.3	1.1 ± 0.3	0.341
INR ^2^	1.24 ± 0.44	1.18 ± 0.0.36	1.26 ± 0.48	0.009 *
EF (%) ^3^	52 ± 10	52 ± 10	52 ± 10	0.561

Continuous values are expressed as mean ± standard deviation. Categorial values are expressed as number and percentage. TIA: transient ischemic attack, CAD: coronary artery disease, PAD: peripheral artery disease, Hb: hemoglobin, BMI: Body Mass Index; GFR: Glomerular Filtration Rate, INR: international normalized ratio, EF: ejection fraction. ^1^: drop out, *n* = 6; ^2^: drop out *n* = 1; ^3^: drop out *n* = 30. * Statistically significant results, *p* < 0.05.

**Table 2:** Type of arrhythmia.

	Total *n* = 479	Conventional *n* = 159	US *n* = 320	*p*-Value
AF (paroxysmal)	107 (22.3%)	43 (27%)	64 (20%)	0.081
AF (persistent)	186 (38.8%)	77 (28.4%)	109 (34.1%)	0.002 *
Atrial tachycardia (AT; right and left atrial)	140 (29.2%)	25 (15.7%)	115 (35.9%)	<0.001 *
Typical atrial flutter	17 (3.5%)	3 (1.9%)	14 (4.4%)	0.166
Premature ventricular contraction (PVC)	14 (2.9%)	6 (3.8%)	8 (2.5%)	0.565
Ventricular tachycardia (VT)	14 (2.9%)	5 (3.1%)	9 (2.8%)	0.782

Categorial values are expressed as number and percentage. AF: atrial fibrillation. * Statistically significant results (*p* < 0.05).

**Table 3:** Oral anticoagulation and antiplatelet agents before the procedure.

Oral Anticoagulation	Total *n* = 479	Conventional *n* = 159	US *n* = 320	*p*-Value
Vitamin K antagonists	37 (7.7%)	8 (5%)	29 (9%)	
Phenprocoumon	35 (7.3%)	8 (5%)	27 (8.4%)	0.177
Warfarine	2 (0.4%)	0 (0%)	2 (0.6%)	1.000
Direct oral anticoagulants	442 (92.3%)	151 (95.0%)	291 (91%)	
Apixaban	278 (58%)	106 (66.7%)	172 (53.8%)	0.007 *
Rivaroxaban	74 (15.4%)	17 (10.7%)	57 (17.8%)	0.042 *
Edoxaban	72 (15%)	23 (14.5%)	49 (15.3%)	0.807
Dabigatran	18 (3.8%)	5 (3.1%)	13 (4.1%)	0.619
Additional antiplatelet therapy	32 (6.7%)	11 (6.9%)	21 (6.6%)	0.883
Acetylsalicylic acid (ASA)	22 (4.6%)	5 (3.1%)	17 (5.3%)	0.286
Clopidogrel	10 (2.1%)	6 (3.8%)	4 (1.3%)	0.09

Categorial values are expressed as number and percentage. * Statistically significant results (*p* < 0.05).

**Table 4:** Procedural Data.

Procedural Data	Total *n* = 479	Conventional *n* = 159	US *n* = 320	*p*-Value
First ablation	219 (45.7%)	76 (47.8%)	143 (44.7%)	0.520
Additional arterial puncture	69 (14.4%)	25 (15.7%)	44 (18.3%)	0.582
Procedure duration (min) ^1^	129.4 ± 53.5	141.7 ± 60.8	123.2 ± 48.4	0.002 *
RF duration (min) ^2^	21.7 ± 14.3	24.8 ± 16.7	20.1 ± 12.7	0.006 *
Hb drop after procedure (mg/dL)	1.08 ± 0.92	1.14 ± 1.00	1.10 ± 0.90	0.374
ACTmin ^3^ [sec]	164 ± 37	159 ± 32	166 ± 38	0.014 *
ACTmax ^3^ [sec]	371 ± 55	364 ± 50	375 ± 58	0.002 *
ACTmean ^3^ [sec]	302 ± 44	299 ± 40	304 ± 46	0.229
Heparine dose ^4^ (IU)	15,726 ± 5386	17,287 ± 5764	14,382 ± 5233	<0.001 *

RF duration: radiofrequency current duration; Hb: hemoglobin; ACT: activated clotting time; ^1^: drop out, *n* = 11; ^2^: drop out, *n* = 14; ^3^: *n* = 9; ^4^: drop out, *n* = 1; * statistically significant results (*p* < 0.05).

**Table 5:** Primary endpoint results: Peri-/postprocedural vascular access complications within 48 h post-procedure and risk reduction by US.

	Total *n* = 479	Conventional *n* = 159	US *n* = 320	*p*-Value	OR	95%-CI
Total	37 (7.7%)	17 (10.7%)	20 (6.3%)	0.086		
Hematoma >5 cm	18 (3.8%)	10 (6.3%)	8 (2.5%)	0.040 *	0.382	0.148–0.988
AV fistula	23 (4.8%)	11 (6.9%)	12 (3.8%)	0.127	0.524	0.226–1.216
AV fistulas with surgical intervention	2 (0.4%)	1 (0.6%)	1 (0.3%)			
Pseudoaneurysm	8 (1.7%)	6 (3.8%)	2 (0.6%)	0.018 *	0.160	0.032–0.804
Pseudoaneurysm with intervention	0					
Retroperitoneal hematoma	2 (0.4%)	1 (0.6%)	1 (0.3%)	0.554	2.019	0.129–32.491

Continuous values are expressed as mean ± standard deviation. Categorial values are expressed as number and percentage. AV fistula: arteriovenous fistula; * statistically significant results (*p* < 0.05).

**Table 6:** Peri-/postprocedural in-hospital thromboembolic and other complications.

Complications	Total *n* = 479	Conventional *n* = 159	US *n* = 320	*p*-Value
Periprocedural thromboembolic complications	2 (0.3%)	1 (0.5%)	1 (0.3%)	0.554
TIA	1 (0.2%)	1 (0.6%)	0	1.00
Apoplexy	1 (0.2%)	0	1 (0.3%)	1.00
Pericardial effusion without tamponade (>5 mm)	33 (6.0%)	9 (4%)	24 (6%)	0.567
Cardiac tamponade	1 (0.2%)	0	1 (0.3%)	1.000
Dysphagia	1 (0.2%)	1 (0.5%)	0	0.332
Pulmonary vein stenosis	0	0	0	
CPR	2 (0.2%)	1 (0.5%)	1 (0.3%)	

CPR: cardiopulmonary resuscitation.

**Table 7:** Subgroup analysis of risk reduction using US in patients with BMI > 30 kg/m^2^.

	OR	95% CI
Hematoma >5 cm	0.051	0.000–0.466
Pseudoaneurysm	0.051	0.000–0.466
Total vascular access compl.	0.138	0.027–0.659
AV fistulas	0.229	0.043–1.228
Retroperitoneal hematoma	2.34	0.113–15.623

## Data Availability

Not applicable.

## Supplementary Materials

The following supporting information can be downloaded at: https://www.mdpi.com/article/10.3390/jcm11226766/s1, Table S1: Risk factors for vascular access complications; Table S2: Risk factor age >70 years for vascular access complications; Table S3: Risk factor female sex.
