# Supplementary material for: Ultrasound-Guided Access Reduces Vascular Complications in Patients Undergoing Catheter Ablation for Cardiac Arrhythmias
Source: J Clin Med. 2022 Nov 15;11(22):6766. doi: 10.3390/jcm11226766 (PMC9696936; doi:10.3390/jcm11226766)
Supplement: Supplementary file 1 [file jcm-11-06766-s001.zip › jcm-1981212-supplementary.pdf]

Supplemental Table S1: Risk factors for vascular access complications

AV-Fistula

	**OR**	**P-value**	**95%-CI**
Vascular disease	3.1	0.007	**1.365–7.457**
Ventricular ablation	12.7	0.003	7.34-28.123

Pseudoaneurysma

	**OR**	**P-value**	**95%-CI**
BMI>30kg/m^2	4.3	0.047	1.019-18.358
Ventricular ablation	5.27	0.048	3.142-8.254

Supplemental Table S2 Risk factor age >70 years for vascular access complications

	**OR**	**p-value**	**95%-CI**
Hematoma >5cm	1.4	0.827	0.601-4.143
Pseudoaneurysm	1.521	0.598	0.392-7.024
AV-fistulas	0.559	0.521	0.389-2.083
Total vascular access complications	1.447	0.689	0.600-2.306

Supplemental Table S3 Risk factor female sex

	**OR**	**p-value**	**95%-CI**
Hematoma >5cm	7.553	0.083	0.645-23.563
Pseudoaneurysm	1.525	0.674	0.3504.180
AV-fistulas	4.384	0.214	0.469-8.371
Total vascular access complications	0.214	0.195	0.164-2.412
